# Supplementary material for: Welfare Issues on Israeli Dairy Farms: Attitudes and Awareness of Farm Workers and Veterinary Practitioners
Source: Animals (Basel). 2021 Jan 24;11(2):294. doi: 10.3390/ani11020294 (PMC7912428; doi:10.3390/ani11020294)

Cows through the Eyes of the Dairy Farmer

Dear dairy farm workers!

We are conducting a survey on the subject of "Cows through the Eyes of the Dairy Farmer".
Our goal is to understand the habitual way in which you, the dairy farmer, view everyday life on the farm, and to learn about your management of the herd, your understanding of their senses, and your approaches to everyday challenges.

This survey is supported by the Milk Dairy Board, with the cooperation of many others in the industry (the Cattle Breeders' Association, the extension service and the Veterinary Services at the Ministry of Agriculture and rural development).

The survey will cover a wide sample of farms, where questionnaires will be given to all of the workers (translated into English, Thai and Arabic as well).

Our aim is to learn from you so that we can establish work interfaces which will benefit all relevant parties.

Survey results would be published without the farms' or peoples' names. Any farm that is interested in viewing its results is welcome to receive them from us.

To get the most from the survey, we would appreciate your full cooperation in answering the questions with the upmost honesty and attention.

Thank you!

The research team.

General Information:

1. Name of the farm:_________________________

2. Worker's age___________

3. Male ☐ Female ☐

4. Job at the cowshed: Dairy farmer ☐ Calf manager ☐ Health manager ☐ Farm manager ☐ Outside guide ☐ Veterinarian ☐ Inseminator ☐

5. Job seniority (years): ___________

6. Education: High school ☐ Professional study/Engineering degree ☐ Academic degree ☐

7. Have you ever been trained to work on a farm? As a farm worker only ☐ In a professional course (for example: "Shaham" extension services, "HaHaklait"). Which course?____________________________

8. Have you ever been instructed on the subject of cattle behavior? Yes ☐ Just a little ☐ Not at all ☐

If you have, in what framework? ____

9. How much do you enjoy your work? (1 – Not at all, 5 – Very much) ☐ 1 ☐ 2 ☐ 3 ☐ 4 ☐ 5

10. How important is the subject of animal welfare to you? (1 – Not at all important, 5 – Very important) ☐ 1 ☐ 2 ☐ 3 ☐ 4 ☐ 5

Please check the answer that **you think** is most suitable:

1. Will the cow's level of dominance affect its position in the herd?
☐ No ☐ Yes ☐ There is no hierarchy in cow herds ☐ I don't know

2. How long does it take for the cow to become acclimated to a new group?

☐ A few minutes ☐ Hours ☐ A day or two ☐ a few days–weeks

3. To what degree does a cow hide pain? (1 – Not at all, 5 – Very much)
☐ 1 ☐ 2 ☐ 3 ☐ 4 ☐ 5

4. Does a person's behavior with calves affect the calves' behavior as adult cows?
☐ Yes ☐ No ☐ I don't know

5. Do you think there is a meaning for the way in which cows get inside the milking parlor?
☐ Yes ☐ No ☐ I don’t know
If yes, please explain. ____

6. Do cows see in the same that way we do?
☐ Yes ☐ No ☐ I don’t know

7. Does the cow hear sounds that we can't?
☐ Yes ☐ No ☐ I don’t know

8. Do you think it is important to give a calf pain killers when removing horn buds (disbudding with the use of caustic paste)?
☐ Yes ☐ No ☐ I don’t know

9. Does a cow remember experiences it had when it was a young calf?
☐ Yes ☐ No ☐ I don’t know

10. Can the milker's behavior "annoy" a cow during milking?
☐ Yes ☐ No ☐ I don’t know

11. Can cows recognize people who treated them negatively or positively in the past?
☐ Yes ☐ No ☐ I don’t know

12. Can the way in which a cow is led to the milking parlor affect the amount of milk it produces during milking?
☐ No ☐ Yes

13. Does high milk production testify to good welfare?
☐ Yes ☐ No ☐ Not necessarily ☐ I don’t know.
Why?____

14. Where, mostly, do you inject the cow with medicine?
☐ In the milking parlor ☐ In a separate yard/shed ☐ Wherever manageable.

15. At what age are your calves transferred to groups (including couples)?
☐ A week at most ☐ Two weeks ☐ A month ☐ At weaning

16. When something is bothering/hurting the cow, will milk production decrease?
☐ Yes ☐ No ☐ Not necessarily ☐ I don’t know

17. How might separating a young cow from the others in the shed and tying it to the inseminator by itself affect its chances of becoming pregnant?
☐ Positively ☐ Negatively ☐ Should not have any effect

18. Does lameness affect milk production?
☐ No ☐ Yes ☐ Not necessarily ☐ I don’t know

19. Do you think cold branding cattle is painful for them?
☐ No ☐ Not necessarily ☐ Yes ☐ I don’t know

20. Which method of rearing during the suckling period is most beneficial for proper development of a healthy calf?
☐ Individual rearing ☐ In couples ☐ In a group ☐ I don't know

Thank you for your cooperation!

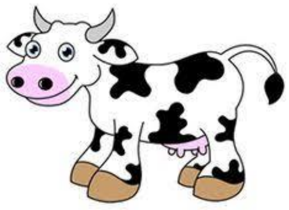

What Do Cows Need?

Veterinary practitioners 'questionnaire regarding cattle's mental needs

General information:

1. **Age: ____**
2. ☐ Female ☐ Male
3. **Seniority (number of years working with cattle): _______**
4. **Have you ever been trained/instructed in the subject of cattle behavior and welfare?**

   ☐ Yes ☐ Briefly ☐ No. If you have, in what framework?____
5. **To what extent do you enjoy your work?** (1 – Not at all, 5 – Very much) ☐ 1 ☐ 2 ☐ 3 ☐ 4 ☐ 5
6. **How important is the subject of animal welfare to you?** (1 – Not important, 5 – Very important) ☐ 1 ☐ 2 ☐ 3 ☐ 4 ☐ 5

Please check the answer that you think is **most correct**:

1. How long does it take a cow to acclimate in a new group?

   ☐ A few minutes ☐ Several hours ☐ A day or two ☐ Several days–weeks
2. How might the separation of a single cow from the others, by leading it from the shed and tying it up alone for insemination, affect its conception?

   ☐ Positively ☐ Negatively ☐ Should not have any effect
3. Does the cow hear things that we cannot hear?

   ☐ Yes ☐ No ☐ I don't know
4. What are your instructions regarding the location where cows should be injected with their medicines?

   ☐ At the milking shed ☐ In a separate yard/shed ☐ Wherever possible
5. Will a cow that is in pain necessarily show a decrease in milk production?

   ☐ No ☐ Not necessarily ☐ Yes ☐ I don't know
6. To what degree does a cow hide its pain? (1 – Not at all, 5 – To a great extent)

   ☐ 1 ☐ 2 ☐ 3 ☐ 4 ☐ 5
7. Is high milk yield an indication of better welfare?

   ☐ Yes ☐ No ☐ Not necessarily ☐ I don't know; and why? ________________
8. Does a human's behavior toward a calf affect that calf's behavior toward humans as an adult cow?

   ☐ Yes ☐ No ☐ I don't know

9. Does a cow remember things that happened to it when it was a young heifer?

☐ Yes ☐ No ☐ I don’t know

10. Do you think that it is essential to give calves painkillers when removing their horn buds (disbudding; with the use of caustic paste)?

☐ Yes ☐ No ☐ I don’t know

11. Do you think painkillers are necessary during cold branding?

☐ Yes ☐ No

12. If so, do you instruct the dairy farmer to use painkillers when branding?

☐ Yes ☐ No

13. Do you think it is necessary to use painkillers after cattle surgery (gastric volvulus, cesarean section, etc.)?

☐ Yes ☐ No ☐ I don’t know

14. Do you think it is necessary to use painkillers for severe lameness?

☐ Yes ☐ No ☐ I don’t know

15. Do you think it is necessary to use painkillers for mastitis?

☐ Yes ☐ No ☐ I don’t know

16. Do cows recognize people who have treated them negatively or positively in the past?

☐ Yes ☐ No ☐ I don’t know

17. What form of rearing contributes the most to the proper development of a healthy calf from infancy to weaning at 2 months of age?

☐ Isolated ☐ In couples ☐ In groups ☐ I don’t know

18. Do cows see as we do?

☐ Yes ☐ No ☐ I don’t know

19. Rank the following options according to their importance to the cow’s welfare: (1 – Most important, 5 – Least important):

☐ Preventing pain and diseases

☐ Providing proper food and drink

☐ Preventing environmental discomfort

☐ Preventing fear and mental distress

☐ Allowing for the expression of natural behavior

20. In the case of an ongoing issue regarding the cow’s welfare, do you usually:

☐ Ignore it ☐ Comment on it ☐ Persist in finding a solution without the help of other professionals ☐ Solve it with the help of other professionals if needed.

Thank you for your cooperation!

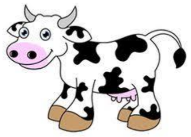

Supplement: Supplementary file 1 [file animals-11-00294-s001.pdf]
